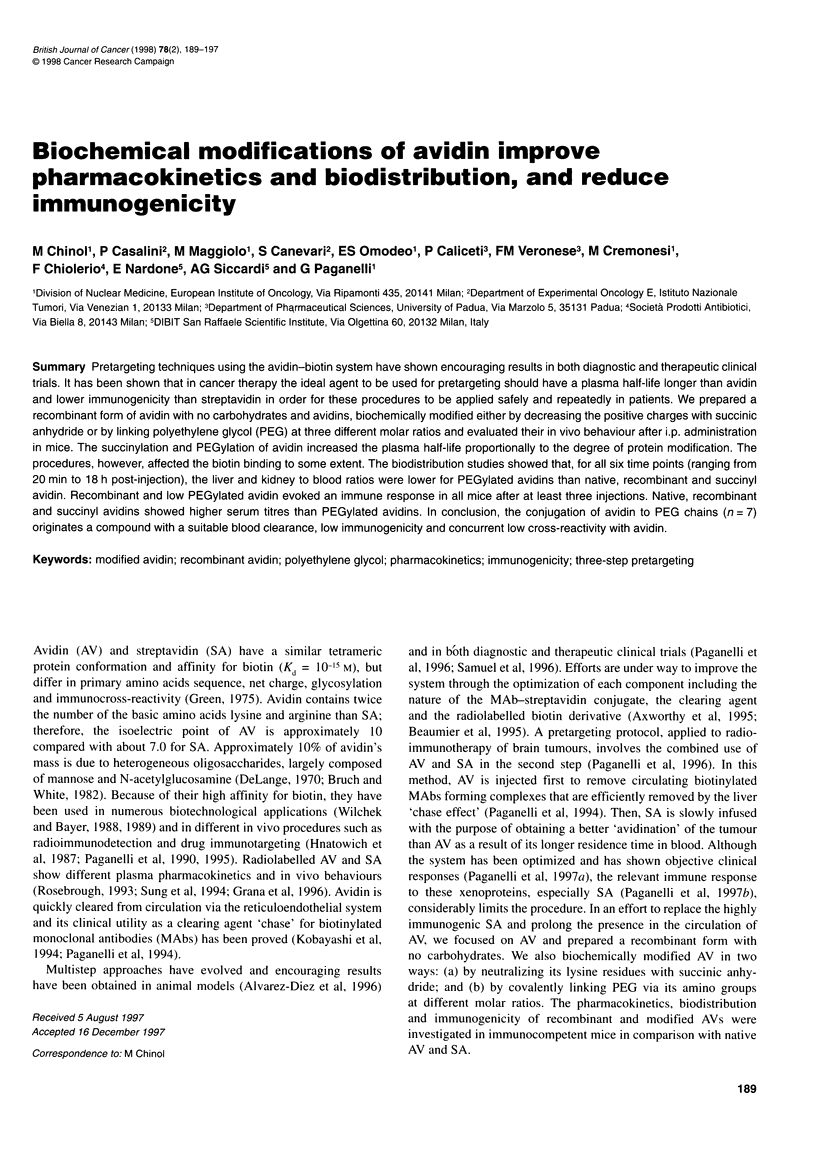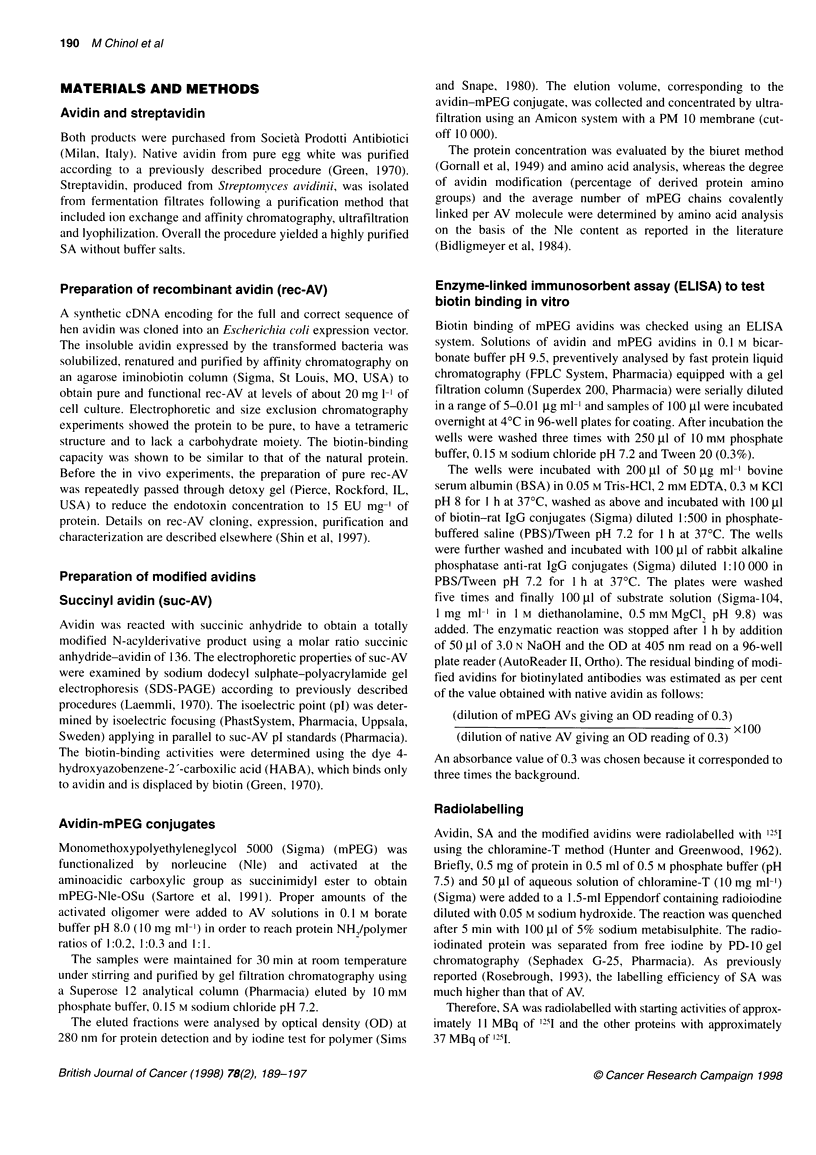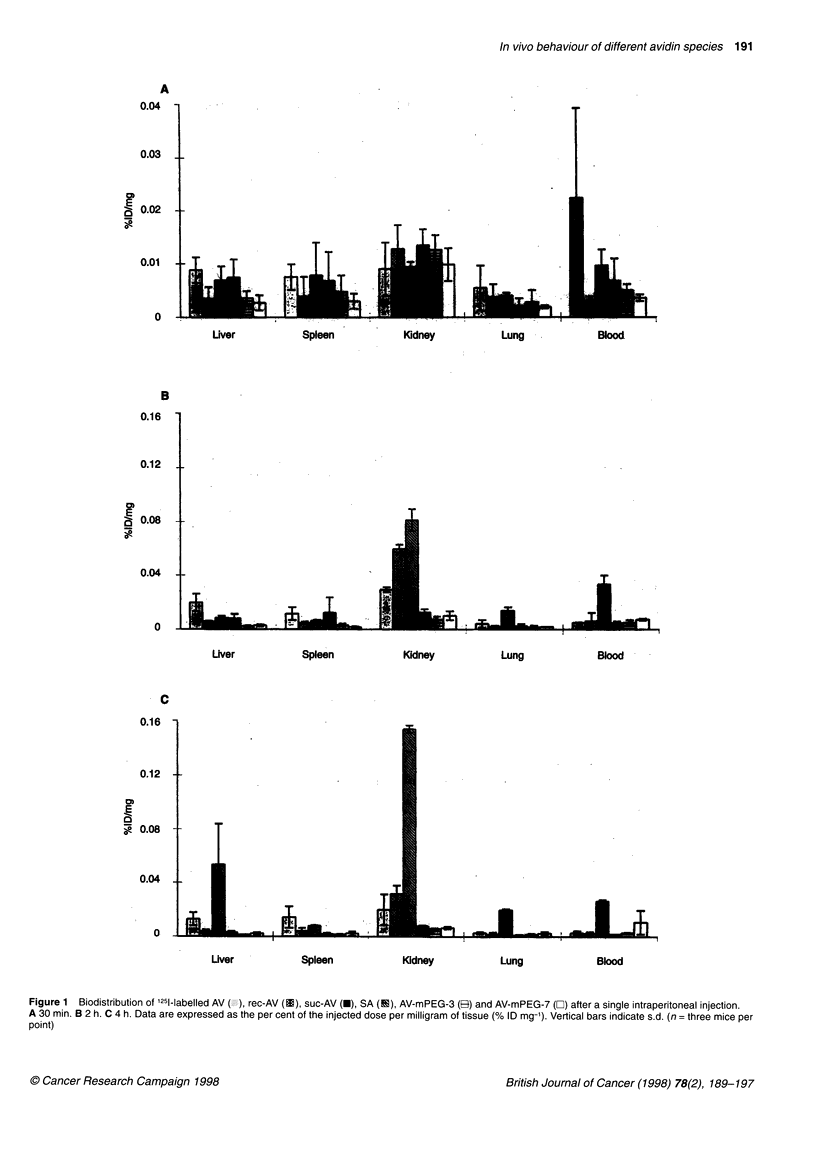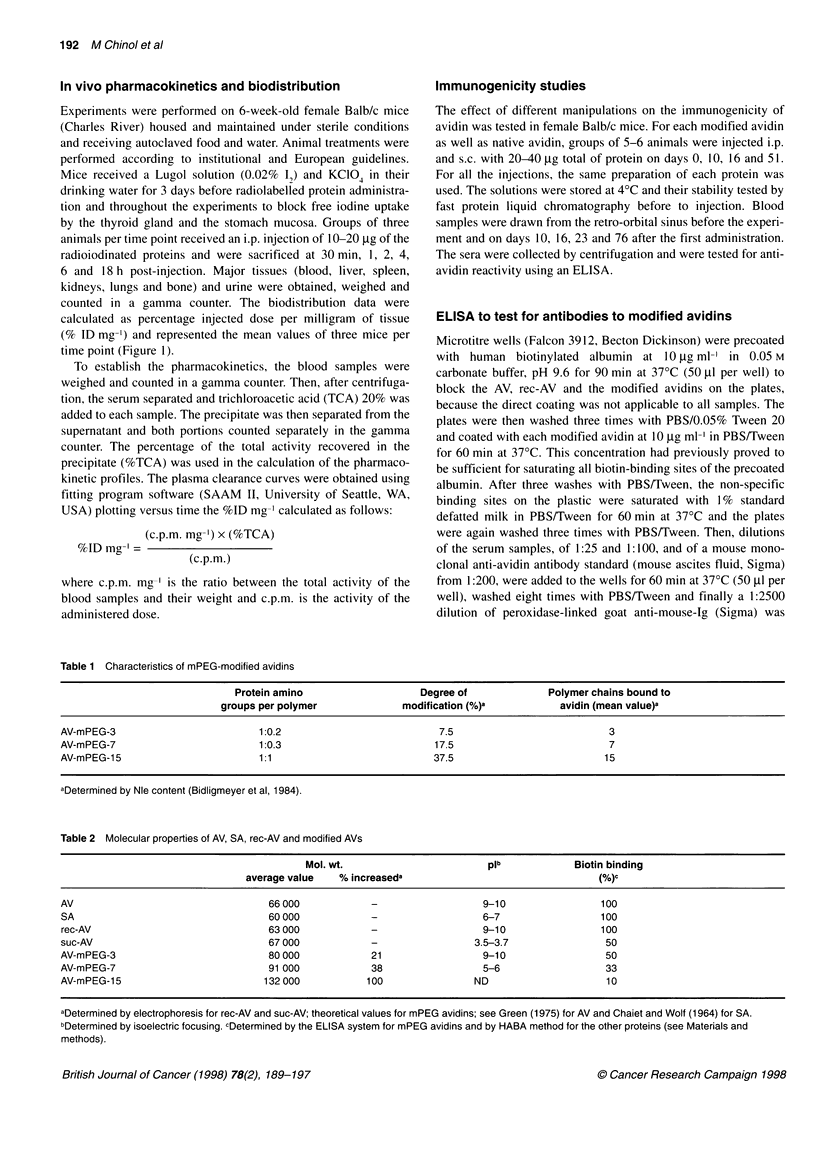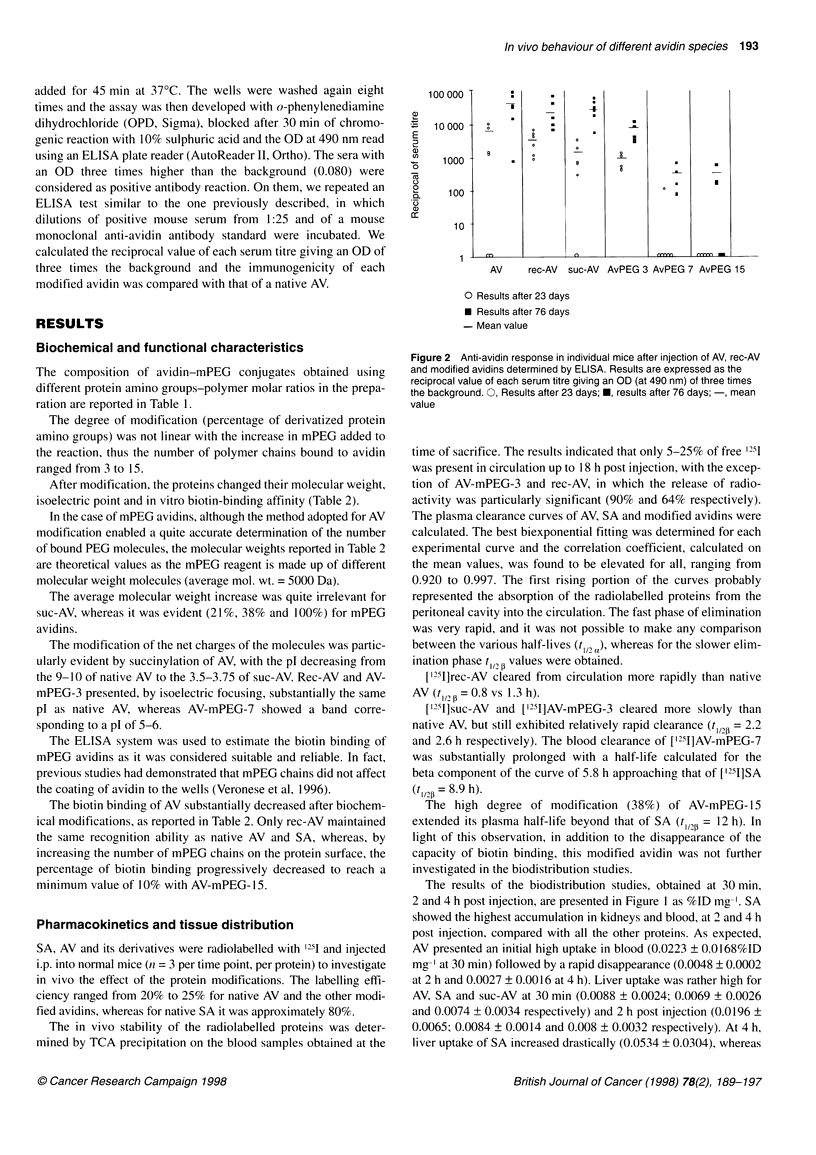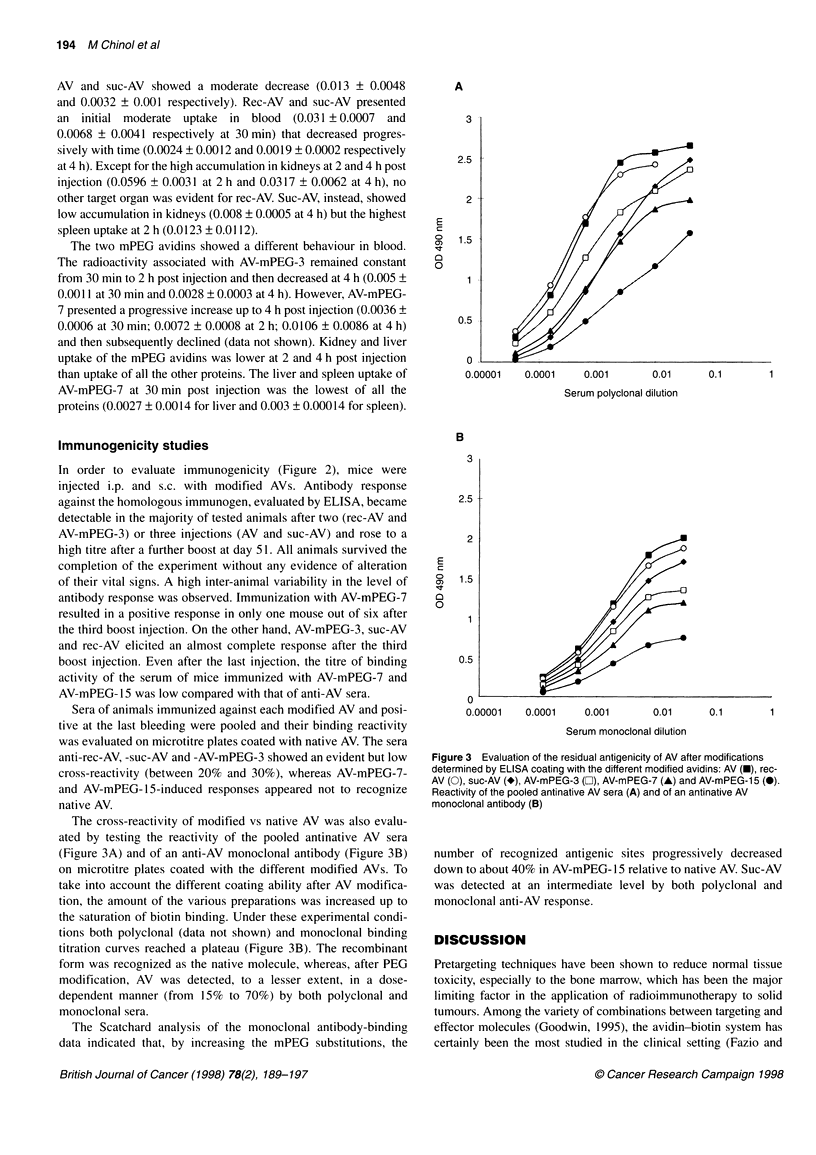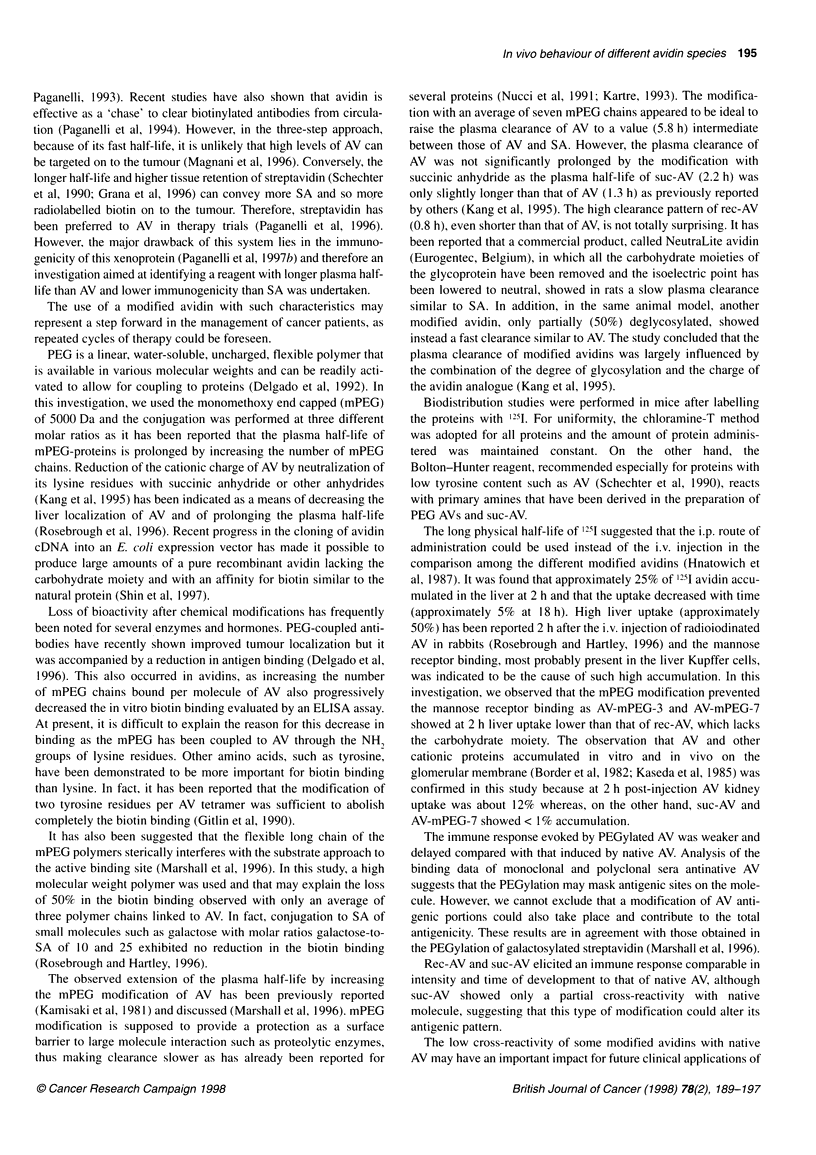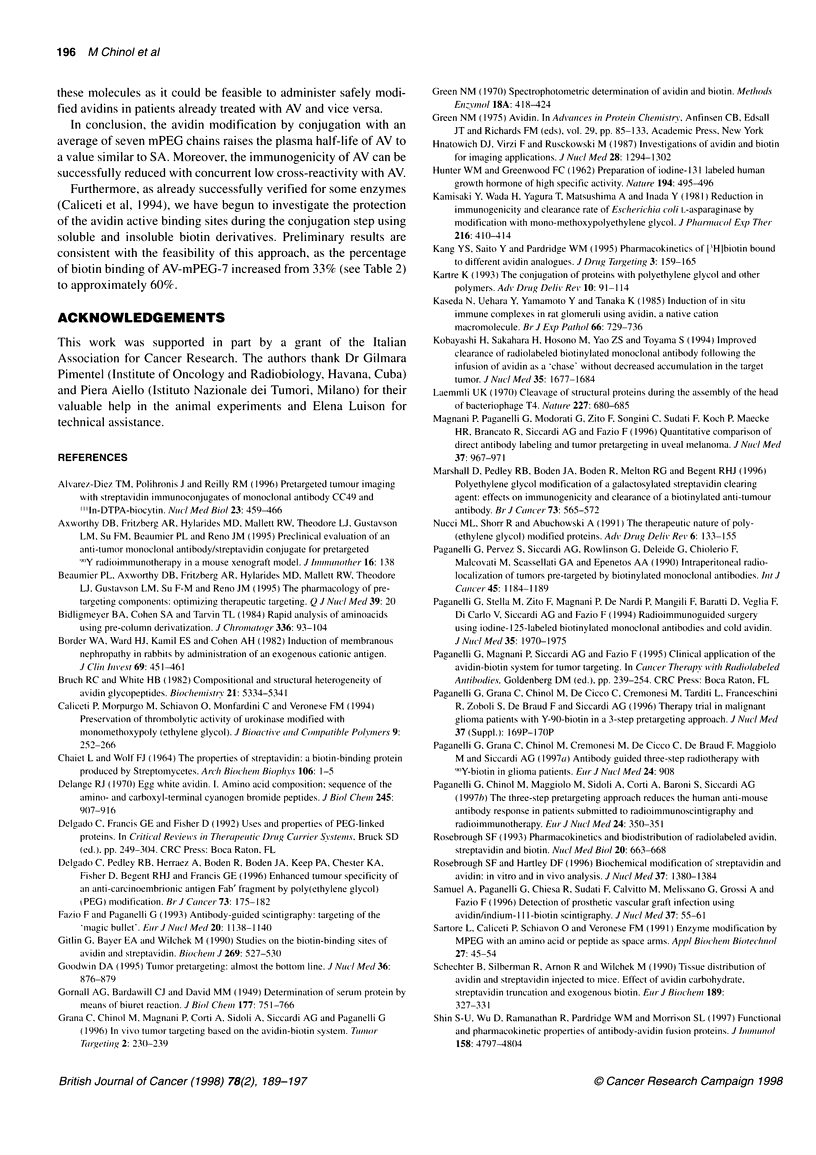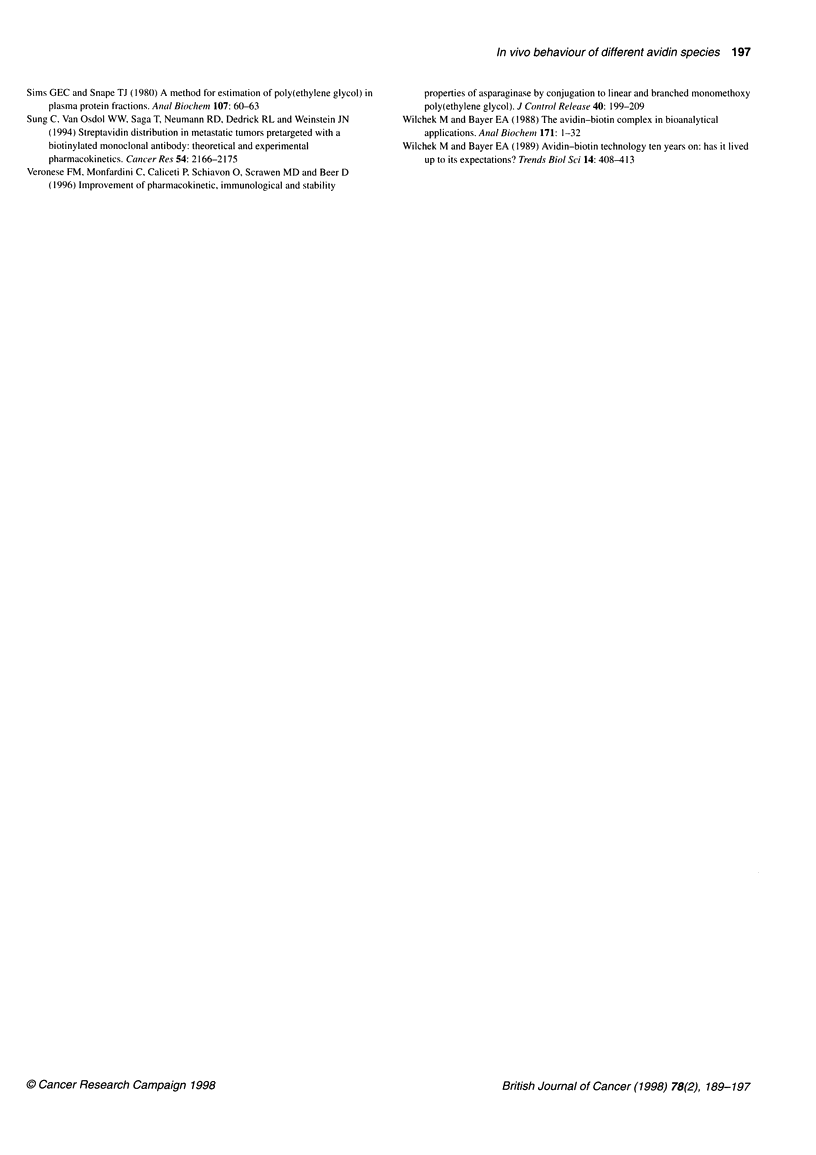# Biochemical modifications of avidin improve pharmacokinetics and biodistribution, and reduce immunogenicity.

**DOI:** 10.1038/bjc.1998.463

**Published:** 1998-07

**Authors:** M. Chinol, P. Casalini, M. Maggiolo, S. Canevari, E. S. Omodeo, P. Caliceti, F. M. Veronese, M. Cremonesi, F. Chiolerio, E. Nardone, A. G. Siccardi, G. Paganelli

**Affiliations:** Division of Nuclear Medicine, European Institute of Oncology, Milan, Italy.

## Abstract

Pretargeting techniques using the avidin-biotin system have shown encouraging results in both diagnostic and therapeutic clinical trials. It has been shown that in cancer therapy the ideal agent to be used for pretargeting should have a plasma half-life longer than avidin and lower immunogenicity than streptavidin in order for these procedures to be applied safely and repeatedly in patients. We prepared a recombinant form of avidin with no carbohydrates and avidins, biochemically modified either by decreasing the positive charges with succinic anhydride or by linking polyethylene glycol (PEG) at three different molar ratios and evaluated their in vivo behaviour after i.p. administration in mice. The succinylation and PEGylation of avidin increased the plasma half-life proportionally to the degree of protein modification. The procedures, however, affected the biotin binding to some extent. The biodistribution studies showed that, for all six time points (ranging from 20 min to 18 h post-injection), the liver and kidney to blood ratios were lower for PEGylated avidins than native, recombinant and succinyl avidin. Recombinant and low PEGylated avidin evoked an immune response in all mice after at least three injections. Native, recombinant and succinyl avidins showed higher serum titres than PEGylated avidins. In conclusion, the conjugation of avidin to PEG chains (n = 7) originates a compound with a suitable blood clearance, low immunogenicity and concurrent low cross-reactivity with avidin.


British Journal of Cancer (1998) 78(2), 189–197
© 1998 Cancer Research Campaign

### Biochemical modifications of avidin improve pharmacokinetics and biodistribution, and reduce immunogenicity

**M Chinol[1], P Casalini[2], M Maggiolo[1], S Canevari[2], ES Omodeo[1], P Caliceti[3], FM Veronese[3], M Cremonesi[1], F Chiolerio[4], E Nardone[5], AG Siccardi[5] and G Paganelli[1]**

[1]Division of Nuclear Medicine, European Institute of Oncology, Via Ripamonti 435, 20141 Milan; [2]Department of Experimental Oncology E, Istituto Nazionale Tumori, Via Venezian 1, 20133 Milan; [3]Department of Pharmaceutical Sciences, University of Padua, Via Marzolo 5, 35131 Padua; [4]Società Prodotti Antibiotici, Via Biella 8, 20143 Milan; [5]DIBIT San Raffaele Scientific Institute, Via Olgettina 60, 20132 Milan, Italy

**Summary** Pretargeting techniques using the avidin–biotin system have shown encouraging results in both diagnostic and therapeutic clinical trials. It has been shown that in cancer therapy the ideal agent to be used for pretargeting should have a plasma half-life longer than avidin and lower immunogenicity than streptavidin in order for these procedures to be applied safely and repeatedly in patients. We prepared a recombinant form of avidin with no carbohydrates and avidins, biochemically modified either by decreasing the positive charges with succinic anhydride or by linking polyethylene glycol (PEG) at three different molar ratios and evaluated their in vivo behaviour after i.p. administration in mice. The succinylation and PEGylation of avidin increased the plasma half-life proportionally to the degree of protein modification. The procedures, however, affected the biotin binding to some extent. The biodistribution studies showed that, for all six time points (ranging from 20 min to 18 h post-injection), the liver and kidney to blood ratios were lower for PEGylated avidins than native, recombinant and succinyl avidin. Recombinant and low PEGylated avidin evoked an immune response in all mice after at least three injections. Native, recombinant and succinyl avidins showed higher serum titres than PEGylated avidins. In conclusion, the conjugation of avidin to PEG chains ($n = 7$) originates a compound with a suitable blood clearance, low immunogenicity and concurrent low cross-reactivity with avidin.

**Keywords:** modified avidin; recombinant avidin; polyethylene glycol; pharmacokinetics; immunogenicity; three-step pretargeting

Avidin (AV) and streptavidin (SA) have a similar tetrameric protein conformation and affinity for biotin ($K_d = 10^{-15}$ M), but differ in primary amino acids sequence, net charge, glycosylation and immunocross-reactivity (Green, 1975). Avidin contains twice the number of the basic amino acids lysine and arginine than SA; therefore, the isoelectric point of AV is approximately 10 compared with about 7.0 for SA. Approximately 10% of avidin's mass is due to heterogeneous oligosaccharides, largely composed of mannose and N-acetylglucosamine (DeLange, 1970; Bruch and White, 1982). Because of their high affinity for biotin, they have been used in numerous biotechnological applications (Wilchek and Bayer, 1988, 1989) and in different in vivo procedures such as radioimmunodetection and drug immunotargeting (Hnatowich et al, 1987; Paganelli et al, 1990, 1995). Radiolabelled AV and SA show different plasma pharmacokinetics and in vivo behaviours (Rosebrough, 1993; Sung et al, 1994; Grana et al, 1996). Avidin is quickly cleared from circulation via the reticuloendothelial system and its clinical utility as a clearing agent 'chase' for biotinylated monoclonal antibodies (MAbs) has been proved (Kobayashi et al, 1994; Paganelli et al, 1994).

Multistep approaches have evolved and encouraging results have been obtained in animal models (Alvarez-Diez et al, 1996) and in both diagnostic and therapeutic clinical trials (Paganelli et al, 1996; Samuel et al, 1996). Efforts are under way to improve the system through the optimization of each component including the nature of the MAb–streptavidin conjugate, the clearing agent and the radiolabelled biotin derivative (Axworthy et al, 1995; Beaumier et al, 1995). A pretargeting protocol, applied to radioimmunotherapy of brain tumours, involves the combined use of AV and SA in the second step (Paganelli et al, 1996). In this method, AV is injected first to remove circulating biotinylated MAbs forming complexes that are efficiently removed by the liver 'chase effect' (Paganelli et al, 1994). Then, SA is slowly infused with the purpose of obtaining a better 'avidination' of the tumour than AV as a result of its longer residence time in blood. Although the system has been optimized and has shown objective clinical responses (Paganelli et al, 1997*a*), the relevant immune response to these xenoproteins, especially SA (Paganelli et al, 1997*b*), considerably limits the procedure. In an effort to replace the highly immunogenic SA and prolong the presence in the circulation of AV, we focused on AV and prepared a recombinant form with no carbohydrates. We also biochemically modified AV in two ways: (a) by neutralizing its lysine residues with succinic anhydride; and (b) by covalently linking PEG via its amino groups at different molar ratios. The pharmacokinetics, biodistribution and immunogenicity of recombinant and modified AVs were investigated in immunocompetent mice in comparison with native AV and SA.

*Received 5 August 1997*
*Accepted 16 December 1997*

*Correspondence to:* M Chinol

### MATERIALS AND METHODS

### Avidin and streptavidin

Both products were purchased from Società Prodotti Antibiotici (Milan, Italy). Native avidin from pure egg white was purified according to a previously described procedure (Green, 1970). Streptavidin, produced from *Streptomyces avidinii*, was isolated from fermentation filtrates following a purification method that included ion exchange and affinity chromatography, ultrafiltration and lyophilization. Overall the procedure yielded a highly purified SA without buffer salts.

### Preparation of recombinant avidin (rec-AV)

A synthetic cDNA encoding for the full and correct sequence of hen avidin was cloned into an *Escherichia coli* expression vector. The insoluble avidin expressed by the transformed bacteria was solubilized, renatured and purified by affinity chromatography on an agarose iminobiotin column (Sigma, St Louis, MO, USA) to obtain pure and functional rec-AV at levels of about 20 mg $l^{-1}$ of cell culture. Electrophoretic and size exclusion chromatography experiments showed the protein to be pure, to have a tetrameric structure and to lack a carbohydrate moiety. The biotin-binding capacity was shown to be similar to that of the natural protein. Before the in vivo experiments, the preparation of pure rec-AV was repeatedly passed through detoxy gel (Pierce, Rockford, IL, USA) to reduce the endotoxin concentration to 15 EU $mg^{-1}$ of protein. Details on rec-AV cloning, expression, purification and characterization are described elsewhere (Shin et al, 1997).

### Preparation of modified avidins

### Succinyl avidin (suc-AV)

Avidin was reacted with succinic anhydride to obtain a totally modified N-acylderivative product using a molar ratio succinic anhydride–avidin of 136. The electrophoretic properties of suc-AV were examined by sodium dodecyl sulphate–polyacrylamide gel electrophoresis (SDS-PAGE) according to previously described procedures (Laemmli, 1970). The isoelectric point (pI) was determined by isoelectric focusing (PhastSystem, Pharmacia, Uppsala, Sweden) applying in parallel to suc-AV pI standards (Pharmacia). The biotin-binding activities were determined using the dye 4-hydroxyazobenzene-2′-carboxilic acid (HABA), which binds only to avidin and is displaced by biotin (Green, 1970).

### Avidin-mPEG conjugates

Monomethoxypolyethyleneglycol 5000 (Sigma) (mPEG) was functionalized by norleucine (Nle) and activated at the aminoacidic carboxylic group as succinimidyl ester to obtain mPEG-Nle-OSu (Sartore et al, 1991). Proper amounts of the activated oligomer were added to AV solutions in 0.1 M borate buffer pH 8.0 (10 mg $ml^{-1}$) in order to reach protein $NH_2$/polymer ratios of 1:0.2, 1:0.3 and 1:1.

The samples were maintained for 30 min at room temperature under stirring and purified by gel filtration chromatography using a Superose 12 analytical column (Pharmacia) eluted by 10 mM phosphate buffer, 0.15 M sodium chloride pH 7.2.

The eluted fractions were analysed by optical density (OD) at 280 nm for protein detection and by iodine test for polymer (Sims and Snape, 1980). The elution volume, corresponding to the avidin–mPEG conjugate, was collected and concentrated by ultrafiltration using an Amicon system with a PM 10 membrane (cut-off 10 000).

The protein concentration was evaluated by the biuret method (Gornall et al, 1949) and amino acid analysis, whereas the degree of avidin modification (percentage of derived protein amino groups) and the average number of mPEG chains covalently linked per AV molecule were determined by amino acid analysis on the basis of the Nle content as reported in the literature (Bidligmeyer et al, 1984).

### Enzyme-linked immunosorbent assay (ELISA) to test biotin binding in vitro

Biotin binding of mPEG avidins was checked using an ELISA system. Solutions of avidin and mPEG avidins in 0.1 M bicarbonate buffer pH 9.5, preventively analysed by fast protein liquid chromatography (FPLC System, Pharmacia) equipped with a gel filtration column (Superdex 200, Pharmacia) were serially diluted in a range of 5–0.01 μg $ml^{-1}$ and samples of 100 μl were incubated overnight at 4°C in 96-well plates for coating. After incubation the wells were washed three times with 250 μl of 10 mM phosphate buffer, 0.15 M sodium chloride pH 7.2 and Tween 20 (0.3%).

The wells were incubated with 200 μl of 50 μg $ml^{-1}$ bovine serum albumin (BSA) in 0.05 M Tris-HCl, 2 mM EDTA, 0.3 M KCl pH 8 for 1 h at 37°C, washed as above and incubated with 100 μl of biotin–rat IgG conjugates (Sigma) diluted 1:500 in phosphate-buffered saline (PBS)/Tween pH 7.2 for 1 h at 37°C. The wells were further washed and incubated with 100 μl of rabbit alkaline phosphatase anti-rat IgG conjugates (Sigma) diluted 1:10 000 in PBS/Tween pH 7.2 for 1 h at 37°C. The plates were washed five times and finally 100 μl of substrate solution (Sigma-104, 1 mg $ml^{-1}$ in 1 M diethanolamine, 0.5 mM $MgCl_2$ pH 9.8) was added. The enzymatic reaction was stopped after 1 h by addition of 50 μl of 3.0 N NaOH and the OD at 405 nm read on a 96-well plate reader (AutoReader II, Ortho). The residual binding of modified avidins for biotinylated antibodies was estimated as per cent of the value obtained with native avidin as follows:

$$\frac{\text{(dilution of mPEG AVs giving an OD reading of 0.3)}}{\text{(dilution of native AV giving an OD reading of 0.3)}} \times 100$$

An absorbance value of 0.3 was chosen because it corresponded to three times the background.

### Radiolabelling

Avidin, SA and the modified avidins were radiolabelled with $^{125}I$ using the chloramine-T method (Hunter and Greenwood, 1962). Briefly, 0.5 mg of protein in 0.5 ml of 0.5 M phosphate buffer (pH 7.5) and 50 μl of aqueous solution of chloramine-T (10 mg $ml^{-1}$) (Sigma) were added to a 1.5-ml Eppendorf containing radioiodine diluted with 0.05 M sodium hydroxide. The reaction was quenched after 5 min with 100 μl of 5% sodium metabisulphite. The radioiodinated protein was separated from free iodine by PD-10 gel chromatography (Sephadex G-25, Pharmacia). As previously reported (Rosebrough, 1993), the labelling efficiency of SA was much higher than that of AV.

Therefore, SA was radiolabelled with starting activities of approximately 11 MBq of $^{125}I$ and the other proteins with approximately 37 MBq of $^{125}I$.

© Cancer Research Campaign 1998

**Figure 1** Biodistribution of $^{125}$I-labelled AV (░), rec-AV (▩), suc-AV (■), SA (▧), AV-mPEG-3 (⊟) and AV-mPEG-7 (□) after a single intraperitoneal injection. **A** 30 min. **B** 2 h. **C** 4 h. Data are expressed as the per cent of the injected dose per milligram of tissue (% ID mg$^{-1}$). Vertical bars indicate s.d. (*n* = three mice per point)

### In vivo pharmacokinetics and biodistribution

Experiments were performed on 6-week-old female Balb/c mice (Charles River) housed and maintained under sterile conditions and receiving autoclaved food and water. Animal treatments were performed according to institutional and European guidelines. Mice received a Lugol solution (0.02% $I_2$) and $KClO_4$ in their drinking water for 3 days before radiolabelled protein administration and throughout the experiments to block free iodine uptake by the thyroid gland and the stomach mucosa. Groups of three animals per time point received an i.p. injection of 10–20 µg of the radioiodinated proteins and were sacrificed at 30 min, 1, 2, 4, 6 and 18 h post-injection. Major tissues (blood, liver, spleen, kidneys, lungs and bone) and urine were obtained, weighed and counted in a gamma counter. The biodistribution data were calculated as percentage injected dose per milligram of tissue (% ID $mg^{-1}$) and represented the mean values of three mice per time point (Figure 1).

To establish the pharmacokinetics, the blood samples were weighed and counted in a gamma counter. Then, after centrifugation, the serum separated and trichloroacetic acid (TCA) 20% was added to each sample. The precipitate was then separated from the supernatant and both portions counted separately in the gamma counter. The percentage of the total activity recovered in the precipitate (%TCA) was used in the calculation of the pharmacokinetic profiles. The plasma clearance curves were obtained using fitting program software (SAAM II, University of Seattle, WA, USA) plotting versus time the %ID $mg^{-1}$ calculated as follows:

$$\%\text{ID mg}^{-1} = \frac{(\text{c.p.m. mg}^{-1}) \times (\%\text{TCA})}{(\text{c.p.m.})}$$

where c.p.m. $mg^{-1}$ is the ratio between the total activity of the blood samples and their weight and c.p.m. is the activity of the administered dose.

### Immunogenicity studies

The effect of different manipulations on the immunogenicity of avidin was tested in female Balb/c mice. For each modified avidin as well as native avidin, groups of 5–6 animals were injected i.p. and s.c. with 20–40 µg total of protein on days 0, 10, 16 and 51. For all the injections, the same preparation of each protein was used. The solutions were stored at 4°C and their stability tested by fast protein liquid chromatography before to injection. Blood samples were drawn from the retro-orbital sinus before the experiment and on days 10, 16, 23 and 76 after the first administration. The sera were collected by centrifugation and were tested for anti-avidin reactivity using an ELISA.

### ELISA to test for antibodies to modified avidins

Microtitre wells (Falcon 3912, Becton Dickinson) were precoated with human biotinylated albumin at 10 µg $ml^{-1}$ in 0.05 M carbonate buffer, pH 9.6 for 90 min at 37°C (50 µl per well) to block the AV, rec-AV and the modified avidins on the plates, because the direct coating was not applicable to all samples. The plates were then washed three times with PBS/0.05% Tween 20 and coated with each modified avidin at 10 µg $ml^{-1}$ in PBS/Tween for 60 min at 37°C. This concentration had previously proved to be sufficient for saturating all biotin-binding sites of the precoated albumin. After three washes with PBS/Tween, the non-specific binding sites on the plastic were saturated with 1% standard defatted milk in PBS/Tween for 60 min at 37°C and the plates were again washed three times with PBS/Tween. Then, dilutions of the serum samples, of 1:25 and 1:100, and of a mouse monoclonal anti-avidin antibody standard (mouse ascites fluid, Sigma) from 1:200, were added to the wells for 60 min at 37°C (50 µl per well), washed eight times with PBS/Tween and finally a 1:2500 dilution of peroxidase-linked goat anti-mouse-Ig (Sigma) was

**Table 1** Characteristics of mPEG-modified avidins

| | Protein amino groups per polymer | Degree of modification (%)[a] | Polymer chains bound to avidin (mean value)[a] |
|---|---|---|---|
| AV-mPEG-3 | 1:0.2 | 7.5 | 3 |
| AV-mPEG-7 | 1:0.3 | 17.5 | 7 |
| AV-mPEG-15 | 1:1 | 37.5 | 15 |

[a]Determined by Nle content (Bidligmeyer et al, 1984).

**Table 2** Molecular properties of AV, SA, rec-AV and modified AVs

| | Mol. wt. average value | Mol. wt. % increased[a] | pI[b] | Biotin binding (%)[c] |
|---|---|---|---|---|
| AV | 66 000 | – | 9–10 | 100 |
| SA | 60 000 | – | 6–7 | 100 |
| rec-AV | 63 000 | – | 9–10 | 100 |
| suc-AV | 67 000 | – | 3.5–3.7 | 50 |
| AV-mPEG-3 | 80 000 | 21 | 9–10 | 50 |
| AV-mPEG-7 | 91 000 | 38 | 5–6 | 33 |
| AV-mPEG-15 | 132 000 | 100 | ND | 10 |

[a]Determined by electrophoresis for rec-AV and suc-AV; theoretical values for mPEG avidins; see Green (1975) for AV and Chaiet and Wolf (1964) for SA. [b]Determined by isoelectric focusing. [c]Determined by the ELISA system for mPEG avidins and by HABA method for the other proteins (see Materials and methods).

 © Cancer Research Campaign 1998

added for 45 min at 37°C. The wells were washed again eight times and the assay was then developed with *o*-phenylenediamine dihydrochloride (OPD, Sigma), blocked after 30 min of chromogenic reaction with 10% sulphuric acid and the OD at 490 nm read using an ELISA plate reader (AutoReader II, Ortho). The sera with an OD three times higher than the background (0.080) were considered as positive antibody reaction. On them, we repeated an ELISA test similar to the one previously described, in which dilutions of positive mouse serum from 1:25 and of a mouse monoclonal anti-avidin antibody standard were incubated. We calculated the reciprocal value of each serum titre giving an OD of three times the background and the immunogenicity of each modified avidin was compared with that of a native AV.

### RESULTS

### Biochemical and functional characteristics

The composition of avidin–mPEG conjugates obtained using different protein amino groups–polymer molar ratios in the preparation are reported in Table 1.

The degree of modification (percentage of derivatized protein amino groups) was not linear with the increase in mPEG added to the reaction, thus the number of polymer chains bound to avidin ranged from 3 to 15.

After modification, the proteins changed their molecular weight, isoelectric point and in vitro biotin-binding affinity (Table 2).

In the case of mPEG avidins, although the method adopted for AV modification enabled a quite accurate determination of the number of bound PEG molecules, the molecular weights reported in Table 2 are theoretical values as the mPEG reagent is made up of different molecular weight molecules (average mol. wt. = 5000 Da).

The average molecular weight increase was quite irrelevant for suc-AV, whereas it was evident (21%, 38% and 100%) for mPEG avidins.

The modification of the net charges of the molecules was particularly evident by succinylation of AV, with the pI decreasing from the 9–10 of native AV to the 3.5–3.75 of suc-AV. Rec-AV and AV-mPEG-3 presented, by isoelectric focusing, substantially the same pI as native AV, whereas AV-mPEG-7 showed a band corresponding to a pI of 5–6.

The ELISA system was used to estimate the biotin binding of mPEG avidins as it was considered suitable and reliable. In fact, previous studies had demonstrated that mPEG chains did not affect the coating of avidin to the wells (Veronese et al, 1996).

The biotin binding of AV substantially decreased after biochemical modifications, as reported in Table 2. Only rec-AV maintained the same recognition ability as native AV and SA, whereas, by increasing the number of mPEG chains on the protein surface, the percentage of biotin binding progressively decreased to reach a minimum value of 10% with AV-mPEG-15.

### Pharmacokinetics and tissue distribution

SA, AV and its derivatives were radiolabelled with $^{125}$I and injected i.p. into normal mice ($n$ = 3 per time point, per protein) to investigate in vivo the effect of the protein modifications. The labelling efficiency ranged from 20% to 25% for native AV and the other modified avidins, whereas for native SA it was approximately 80%.

The in vivo stability of the radiolabelled proteins was determined by TCA precipitation on the blood samples obtained at the time of sacrifice. The results indicated that only 5–25% of free $^{125}$I was present in circulation up to 18 h post injection, with the exception of AV-mPEG-3 and rec-AV, in which the release of radioactivity was particularly significant (90% and 64% respectively). The plasma clearance curves of AV, SA and modified avidins were calculated. The best biexponential fitting was determined for each experimental curve and the correlation coefficient, calculated on the mean values, was found to be elevated for all, ranging from 0.920 to 0.997. The first rising portion of the curves probably represented the absorption of the radiolabelled proteins from the peritoneal cavity into the circulation. The fast phase of elimination was very rapid, and it was not possible to make any comparison between the various half-lives ($t_{1/2\alpha}$), whereas for the slower elimination phase $t_{1/2\beta}$ values were obtained.

[$^{125}$I]rec-AV cleared from circulation more rapidly than native AV ($t_{1/2\beta}$ = 0.8 vs 1.3 h).

[$^{125}$I]suc-AV and [$^{125}$I]AV-mPEG-3 cleared more slowly than native AV, but still exhibited relatively rapid clearance ($t_{1/2\beta}$ = 2.2 and 2.6 h respectively). The blood clearance of [$^{125}$I]AV-mPEG-7 was substantially prolonged with a half-life calculated for the beta component of the curve of 5.8 h approaching that of [$^{125}$I]SA ($t_{1/2\beta}$ = 8.9 h).

The high degree of modification (38%) of AV-mPEG-15 extended its plasma half-life beyond that of SA ($t_{1/2\beta}$ = 12 h). In light of this observation, in addition to the disappearance of the capacity of biotin binding, this modified avidin was not further investigated in the biodistribution studies.

The results of the biodistribution studies, obtained at 30 min, 2 and 4 h post injection, are presented in Figure 1 as %ID mg$^{-1}$. SA showed the highest accumulation in kidneys and blood, at 2 and 4 h post injection, compared with all the other proteins. As expected, AV presented an initial high uptake in blood (0.0223 ± 0.0168%ID mg$^{-1}$ at 30 min) followed by a rapid disappearance (0.0048 ± 0.0002 at 2 h and 0.0027 ± 0.0016 at 4 h). Liver uptake was rather high for AV, SA and suc-AV at 30 min (0.0088 ± 0.0024; 0.0069 ± 0.0026 and 0.0074 ± 0.0034 respectively) and 2 h post injection (0.0196 ± 0.0065; 0.0084 ± 0.0014 and 0.008 ± 0.0032 respectively). At 4 h, liver uptake of SA increased drastically (0.0534 ± 0.0304), whereas

**Figure 2** Anti-avidin response in individual mice after injection of AV, rec-AV and modified avidins determined by ELISA. Results are expressed as the reciprocal value of each serum titre giving an OD (at 490 nm) of three times the background. ○, Results after 23 days; ■, results after 76 days; —, mean value

AV and suc-AV showed a moderate decrease (0.013 ± 0.0048 and 0.0032 ± 0.001 respectively). Rec-AV and suc-AV presented an initial moderate uptake in blood (0.031 ± 0.0007 and 0.0068 ± 0.0041 respectively at 30 min) that decreased progressively with time (0.0024 ± 0.0012 and 0.0019 ± 0.0002 respectively at 4 h). Except for the high accumulation in kidneys at 2 and 4 h post injection (0.0596 ± 0.0031 at 2 h and 0.0317 ± 0.0062 at 4 h), no other target organ was evident for rec-AV. Suc-AV, instead, showed low accumulation in kidneys (0.008 ± 0.0005 at 4 h) but the highest spleen uptake at 2 h (0.0123 ± 0.0112).

The two mPEG avidins showed a different behaviour in blood. The radioactivity associated with AV-mPEG-3 remained constant from 30 min to 2 h post injection and then decreased at 4 h (0.005 ± 0.0011 at 30 min and 0.0028 ± 0.0003 at 4 h). However, AV-mPEG-7 presented a progressive increase up to 4 h post injection (0.0036 ± 0.0006 at 30 min; 0.0072 ± 0.0008 at 2 h; 0.0106 ± 0.0086 at 4 h) and then subsequently declined (data not shown). Kidney and liver uptake of the mPEG avidins was lower at 2 and 4 h post injection than uptake of all the other proteins. The liver and spleen uptake of AV-mPEG-7 at 30 min post injection was the lowest of all the proteins (0.0027 ± 0.0014 for liver and 0.003 ± 0.00014 for spleen).

### Immunogenicity studies

In order to evaluate immunogenicity (Figure 2), mice were injected i.p. and s.c. with modified AVs. Antibody response against the homologous immunogen, evaluated by ELISA, became detectable in the majority of tested animals after two (rec-AV and AV-mPEG-3) or three injections (AV and suc-AV) and rose to a high titre after a further boost at day 51. All animals survived the completion of the experiment without any evidence of alteration of their vital signs. A high inter-animal variability in the level of antibody response was observed. Immunization with AV-mPEG-7 resulted in a positive response in only one mouse out of six after the third boost injection. On the other hand, AV-mPEG-3, suc-AV and rec-AV elicited an almost complete response after the third boost injection. Even after the last injection, the titre of binding activity of the serum of mice immunized with AV-mPEG-7 and AV-mPEG-15 was low compared with that of anti-AV sera.

Sera of animals immunized against each modified AV and positive at the last bleeding were pooled and their binding reactivity was evaluated on microtitre plates coated with native AV. The sera anti-rec-AV, -suc-AV and -AV-mPEG-3 showed an evident but low cross-reactivity (between 20% and 30%), whereas AV-mPEG-7- and AV-mPEG-15-induced responses appeared not to recognize native AV.

The cross-reactivity of modified vs native AV was also evaluated by testing the reactivity of the pooled antinative AV sera (Figure 3A) and of an anti-AV monoclonal antibody (Figure 3B) on microtitre plates coated with the different modified AVs. To take into account the different coating ability after AV modification, the amount of the various preparations was increased up to the saturation of biotin binding. Under these experimental conditions both polyclonal (data not shown) and monoclonal binding titration curves reached a plateau (Figure 3B). The recombinant form was recognized as the native molecule, whereas, after PEG modification, AV was detected, to a lesser extent, in a dose-dependent manner (from 15% to 70%) by both polyclonal and monoclonal sera.

The Scatchard analysis of the monoclonal antibody-binding data indicated that, by increasing the mPEG substitutions, the number of recognized antigenic sites progressively decreased down to about 40% in AV-mPEG-15 relative to native AV. Suc-AV was detected at an intermediate level by both polyclonal and monoclonal anti-AV response.

**Figure 3** Evaluation of the residual antigenicity of AV after modifications determined by ELISA coating with the different modified avidins: AV (■), rec-AV (○), suc-AV (◆), AV-mPEG-3 (□), AV-mPEG-7 (▲) and AV-mPEG-15 (●). Reactivity of the pooled antinative AV sera (**A**) and of an antinative AV monoclonal antibody (**B**)

### DISCUSSION

Pretargeting techniques have been shown to reduce normal tissue toxicity, especially to the bone marrow, which has been the major limiting factor in the application of radioimmunotherapy to solid tumours. Among the variety of combinations between targeting and effector molecules (Goodwin, 1995), the avidin–biotin system has certainly been the most studied in the clinical setting (Fazio and

© Cancer Research Campaign 1998

Paganelli, 1993). Recent studies have also shown that avidin is effective as a 'chase' to clear biotinylated antibodies from circulation (Paganelli et al, 1994). However, in the three-step approach, because of its fast half-life, it is unlikely that high levels of AV can be targeted on to the tumour (Magnani et al, 1996). Conversely, the longer half-life and higher tissue retention of streptavidin (Schechter et al, 1990; Grana et al, 1996) can convey more SA and so more radiolabelled biotin on to the tumour. Therefore, streptavidin has been preferred to AV in therapy trials (Paganelli et al, 1996). However, the major drawback of this system lies in the immunogenicity of this xenoprotein (Paganelli et al, 1997*b*) and therefore an investigation aimed at identifying a reagent with longer plasma half-life than AV and lower immunogenicity than SA was undertaken.

The use of a modified avidin with such characteristics may represent a step forward in the management of cancer patients, as repeated cycles of therapy could be foreseen.

PEG is a linear, water-soluble, uncharged, flexible polymer that is available in various molecular weights and can be readily activated to allow for coupling to proteins (Delgado et al, 1992). In this investigation, we used the monomethoxy end capped (mPEG) of 5000 Da and the conjugation was performed at three different molar ratios as it has been reported that the plasma half-life of mPEG-proteins is prolonged by increasing the number of mPEG chains. Reduction of the cationic charge of AV by neutralization of its lysine residues with succinic anhydride or other anhydrides (Kang et al, 1995) has been indicated as a means of decreasing the liver localization of AV and of prolonging the plasma half-life (Rosebrough et al, 1996). Recent progress in the cloning of avidin cDNA into an *E. coli* expression vector has made it possible to produce large amounts of a pure recombinant avidin lacking the carbohydrate moiety and with an affinity for biotin similar to the natural protein (Shin et al, 1997).

Loss of bioactivity after chemical modifications has frequently been noted for several enzymes and hormones. PEG-coupled antibodies have recently shown improved tumour localization but it was accompanied by a reduction in antigen binding (Delgado et al, 1996). This also occurred in avidins, as increasing the number of mPEG chains bound per molecule of AV also progressively decreased the in vitro biotin binding evaluated by an ELISA assay. At present, it is difficult to explain the reason for this decrease in binding as the mPEG has been coupled to AV through the $NH_2$ groups of lysine residues. Other amino acids, such as tyrosine, have been demonstrated to be more important for biotin binding than lysine. In fact, it has been reported that the modification of two tyrosine residues per AV tetramer was sufficient to abolish completely the biotin binding (Gitlin et al, 1990).

It has also been suggested that the flexible long chain of the mPEG polymers sterically interferes with the substrate approach to the active binding site (Marshall et al, 1996). In this study, a high molecular weight polymer was used and that may explain the loss of 50% in the biotin binding observed with only an average of three polymer chains linked to AV. In fact, conjugation to SA of small molecules such as galactose with molar ratios galactose-to-SA of 10 and 25 exhibited no reduction in the biotin binding (Rosebrough and Hartley, 1996).

The observed extension of the plasma half-life by increasing the mPEG modification of AV has been previously reported (Kamisaki et al, 1981) and discussed (Marshall et al, 1996). mPEG modification is supposed to provide a protection as a surface barrier to large molecule interaction such as proteolytic enzymes, thus making clearance slower as has already been reported for several proteins (Nucci et al, 1991; Kartre, 1993). The modification with an average of seven mPEG chains appeared to be ideal to raise the plasma clearance of AV to a value (5.8 h) intermediate between those of AV and SA. However, the plasma clearance of AV was not significantly prolonged by the modification with succinic anhydride as the plasma half-life of suc-AV (2.2 h) was only slightly longer than that of AV (1.3 h) as previously reported by others (Kang et al, 1995). The high clearance pattern of rec-AV (0.8 h), even shorter than that of AV, is not totally surprising. It has been reported that a commercial product, called NeutraLite avidin (Eurogentec, Belgium), in which all the carbohydrate moieties of the glycoprotein have been removed and the isoelectric point has been lowered to neutral, showed in rats a slow plasma clearance similar to SA. In addition, in the same animal model, another modified avidin, only partially (50%) deglycosylated, showed instead a fast clearance similar to AV. The study concluded that the plasma clearance of modified avidins was largely influenced by the combination of the degree of glycosylation and the charge of the avidin analogue (Kang et al, 1995).

Biodistribution studies were performed in mice after labelling the proteins with $^{125}$I. For uniformity, the chloramine-T method was adopted for all proteins and the amount of protein administered was maintained constant. On the other hand, the Bolton–Hunter reagent, recommended especially for proteins with low tyrosine content such as AV (Schechter et al, 1990), reacts with primary amines that have been derived in the preparation of PEG AVs and suc-AV.

The long physical half-life of $^{125}$I suggested that the i.p. route of administration could be used instead of the i.v. injection in the comparison among the different modified avidins (Hnatowich et al, 1987). It was found that approximately 25% of $^{125}$I avidin accumulated in the liver at 2 h and that the uptake decreased with time (approximately 5% at 18 h). High liver uptake (approximately 50%) has been reported 2 h after the i.v. injection of radioiodinated AV in rabbits (Rosebrough and Hartley, 1996) and the mannose receptor binding, most probably present in the liver Kupffer cells, was indicated to be the cause of such high accumulation. In this investigation, we observed that the mPEG modification prevented the mannose receptor binding as AV-mPEG-3 and AV-mPEG-7 showed at 2 h liver uptake lower than that of rec-AV, which lacks the carbohydrate moiety. The observation that AV and other cationic proteins accumulated in vitro and in vivo on the glomerular membrane (Border et al, 1982; Kaseda et al, 1985) was confirmed in this study because at 2 h post-injection AV kidney uptake was about 12% whereas, on the other hand, suc-AV and AV-mPEG-7 showed $< 1\%$ accumulation.

The immune response evoked by PEGylated AV was weaker and delayed compared with that induced by native AV. Analysis of the binding data of monoclonal and polyclonal sera antinative AV suggests that the PEGylation may mask antigenic sites on the molecule. However, we cannot exclude that a modification of AV antigenic portions could also take place and contribute to the total antigenicity. These results are in agreement with those obtained in the PEGylation of galactosylated streptavidin (Marshall et al, 1996).

Rec-AV and suc-AV elicited an immune response comparable in intensity and time of development to that of native AV, although suc-AV showed only a partial cross-reactivity with native molecule, suggesting that this type of modification could alter its antigenic pattern.

The low cross-reactivity of some modified avidins with native AV may have an important impact for future clinical applications of

© Cancer Research Campaign 1998

these molecules as it could be feasible to administer safely modified avidins in patients already treated with AV and vice versa.

In conclusion, the avidin modification by conjugation with an average of seven mPEG chains raises the plasma half-life of AV to a value similar to SA. Moreover, the immunogenicity of AV can be successfully reduced with concurrent low cross-reactivity with AV.

Furthermore, as already successfully verified for some enzymes (Caliceti et al, 1994), we have begun to investigate the protection of the avidin active binding sites during the conjugation step using soluble and insoluble biotin derivatives. Preliminary results are consistent with the feasibility of this approach, as the percentage of biotin binding of AV-mPEG-7 increased from 33% (see Table 2) to approximately 60%.

### ACKNOWLEDGEMENTS

This work was supported in part by a grant of the Italian Association for Cancer Research. The authors thank Dr Gilmara Pimentel (Institute of Oncology and Radiobiology, Havana, Cuba) and Piera Aiello (Istituto Nazionale dei Tumori, Milano) for their valuable help in the animal experiments and Elena Luison for technical assistance.

### REFERENCES

Alvarez-Diez TM, Polihronis J and Reilly RM (1996) Pretargeted tumour imaging with streptavidin immunoconjugates of monoclonal antibody CC49 and $^{111}$In-DTPA-biocytin. *Nucl Med Biol* **23**: 459–466

Axworthy DB, Fritzberg AR, Hylarides MD, Mallett RW, Theodore LJ, Gustavson LM, Su FM, Beaumier PL and Reno JM (1995) Preclinical evaluation of an anti-tumor monoclonal antibody/streptavidin conjugate for pretargeted $^{90}$Y radioimmunotherapy in a mouse xenograft model. *J Immunother* **16**: 138

Beaumier PL, Axworthy DB, Fritzberg AR, Hylarides MD, Mallett RW, Theodore LJ, Gustavson LM, Su F-M and Reno JM (1995) The pharmacology of pretargeting components: optimizing therapeutic targeting. *Q J Nucl Med* **39**: 20

Bidligmeyer BA, Cohen SA and Tarvin TL (1984) Rapid analysis of aminoacids using pre-column derivatization. *J Chromatogr* **336**: 93–104

Border WA, Ward HJ, Kamil ES and Cohen AH (1982) Induction of membranous nephropathy in rabbits by administration of an exogenous cationic antigen. *J Clin Invest* **69**: 451–461

Bruch RC and White HB (1982) Compositional and structural heterogeneity of avidin glycopeptides. *Biochemistry* **21**: 5334–5341

Caliceti P, Morpurgo M, Schiavon O, Monfardini C and Veronese FM (1994) Preservation of thrombolytic activity of urokinase modified with monomethoxypoly (ethylene glycol). *J Bioactive and Compatible Polymers* **9**: 252–266

Chaiet L and Wolf FJ (1964) The properties of streptavidin: a biotin-binding protein produced by Streptomycetes. *Arch Biochem Biophys* **106**: 1–5

Delange RJ (1970) Egg white avidin. I. Amino acid composition; sequence of the amino- and carboxyl-terminal cyanogen bromide peptides. *J Biol Chem* **245**: 907–916

Delgado C, Francis GE and Fisher D (1992) Uses and properties of PEG-linked proteins. In *Critical Reviews in Therapeutic Drug Carrier Systems*, Bruck SD (ed.), pp. 249–304. CRC Press: Boca Raton, FL

Delgado C, Pedley RB, Herraez A, Boden R, Boden JA, Keep PA, Chester KA, Fisher D, Begent RHJ and Francis GE (1996) Enhanced tumour specificity of an anti-carcinoembrionic antigen Fab′ fragment by poly(ethylene glycol) (PEG) modification. *Br J Cancer* **73**: 175–182

Fazio F and Paganelli G (1993) Antibody-guided scintigraphy: targeting of the 'magic bullet'. *Eur J Nucl Med* **20**: 1138–1140

Gitlin G, Bayer EA and Wilchek M (1990) Studies on the biotin-binding sites of avidin and streptavidin. *Biochem J* **269**: 527–530

Goodwin DA (1995) Tumor pretargeting: almost the bottom line. *J Nucl Med* **36**: 876–879

Gornall AG, Bardawill CJ and David MM (1949) Determination of serum protein by means of biuret reaction. *J Biol Chem* **177**: 751–766

Grana C, Chinol M, Magnani P, Corti A, Sidoli A, Siccardi AG and Paganelli G (1996) In vivo tumor targeting based on the avidin-biotin system. *Tumor Targeting* **2**: 230–239

Green NM (1970) Spectrophotometric determination of avidin and biotin. *Methods Enzymol* **18A**: 418–424

Green NM (1975) Avidin. In *Advances in Protein Chemistry*, Anfinsen CB, Edsall JT and Richards FM (eds), vol. 29, pp. 85–133, Academic Press, New York

Hnatowich DJ, Virzi F and Rusckowski M (1987) Investigations of avidin and biotin for imaging applications. *J Nucl Med* **28**: 1294–1302

Hunter WM and Greenwood FC (1962) Preparation of iodine-131 labeled human growth hormone of high specific activity. *Nature* **194**: 495–496

Kamisaki Y, Wada H, Yagura T, Matsushima A and Inada Y (1981) Reduction in immunogenicity and clearance rate of *Escherichia coli* L-asparaginase by modification with mono-methoxypolyethylene glycol. *J Pharmacol Exp Ther* **216**: 410–414

Kang YS, Saito Y and Pardridge WM (1995) Pharmacokinetics of [$^{3}$H]biotin bound to different avidin analogues. *J Drug Targeting* **3**: 159–165

Kartre K (1993) The conjugation of proteins with polyethylene glycol and other polymers. *Adv Drug Deliv Rev* **10**: 91–114

Kaseda N, Uehara Y, Yamamoto Y and Tanaka K (1985) Induction of in situ immune complexes in rat glomeruli using avidin, a native cation macromolecule. *Br J Exp Pathol* **66**: 729–736

Kobayashi H, Sakahara H, Hosono M, Yao ZS and Toyama S (1994) Improved clearance of radiolabeled biotinylated monoclonal antibody following the infusion of avidin as a 'chase' without decreased accumulation in the target tumor. *J Nucl Med* **35**: 1677–1684

Laemmli UK (1970) Cleavage of structural proteins during the assembly of the head of bacteriophage T4. *Nature* **227**: 680–685

Magnani P, Paganelli G, Modorati G, Zito F, Songini C, Sudati F, Koch P, Maecke HR, Brancato R, Siccardi AG and Fazio F (1996) Quantitative comparison of direct antibody labeling and tumor pretargeting in uveal melanoma. *J Nucl Med* **37**: 967–971

Marshall D, Pedley RB, Boden JA, Boden R, Melton RG and Begent RHJ (1996) Polyethylene glycol modification of a galactosylated streptavidin clearing agent: effects on immunogenicity and clearance of a biotinylated anti-tumour antibody. *Br J Cancer* **73**: 565–572

Nucci ML, Shorr R and Abuchowski A (1991) The therapeutic nature of poly-(ethylene glycol) modified proteins. *Adv Drug Deliv Rev* **6**: 133–155

Paganelli G, Pervez S, Siccardi AG, Rowlinson G, Deleide G, Chiolerio F, Malcovati M, Scassellati GA and Epenetos AA (1990) Intraperitoneal radio-localization of tumors pre-targeted by biotinylated monoclonal antibodies. *Int J Cancer* **45**: 1184–1189

Paganelli G, Stella M, Zito F, Magnani P, De Nardi P, Mangili F, Baratti D, Veglia F, Di Carlo V, Siccardi AG and Fazio F (1994) Radioimmunoguided surgery using iodine-125-labeled biotinylated monoclonal antibodies and cold avidin. *J Nucl Med* **35**: 1970–1975

Paganelli G, Magnani P, Siccardi AG and Fazio F (1995) Clinical application of the avidin-biotin system for tumor targeting. In *Cancer Therapy with Radiolabeled Antibodies*, Goldenberg DM (ed.), pp. 239–254. CRC Press: Boca Raton, FL

Paganelli G, Grana C, Chinol M, De Cicco C, Cremonesi M, Tarditi L, Franceschini R, Zoboli S, De Braud F and Siccardi AG (1996) Therapy trial in malignant glioma patients with Y-90-biotin in a 3-step pretargeting approach. *J Nucl Med* **37** (Suppl.): 169P–170P

Paganelli G, Grana C, Chinol M, Cremonesi M, De Cicco C, De Braud F, Maggiolo M and Siccardi AG (1997*a*) Antibody guided three-step radiotherapy with $^{90}$Y-biotin in glioma patients. *Eur J Nucl Med* **24**: 908

Paganelli G, Chinol M, Maggiolo M, Sidoli A, Corti A, Baroni S, Siccardi AG (1997*b*) The three-step pretargeting approach reduces the human anti-mouse antibody response in patients submitted to radioimmunoscintigraphy and radioimmunotherapy. *Eur J Nucl Med* **24**: 350–351

Rosebrough SF (1993) Pharmacokinetics and biodistribution of radiolabeled avidin, streptavidin and biotin. *Nucl Med Biol* **20**: 663–668

Rosebrough SF and Hartley DF (1996) Biochemical modification of streptavidin and avidin: in vitro and in vivo analysis. *J Nucl Med* **37**: 1380–1384

Samuel A, Paganelli G, Chiesa R, Sudati F, Calvitto M, Melissano G, Grossi A and Fazio F (1996) Detection of prosthetic vascular graft infection using avidin/indium-111-biotin scintigraphy. *J Nucl Med* **37**: 55–61

Sartore L, Caliceti P, Schiavon O and Veronese FM (1991) Enzyme modification by MPEG with an amino acid or peptide as space arms. *Appl Biochem Biotechnol* **27**: 45–54

Schechter B, Silberman R, Arnon R and Wilchek M (1990) Tissue distribution of avidin and streptavidin injected to mice. Effect of avidin carbohydrate, streptavidin truncation and exogenous biotin. *Eur J Biochem* **189**: 327–331

Shin S-U, Wu D, Ramanathan R, Pardridge WM and Morrison SL (1997) Functional and pharmacokinetic properties of antibody-avidin fusion proteins. *J Immunol* **158**: 4797–4804

 © Cancer Research Campaign 1998

Sims GEC and Snape TJ (1980) A method for estimation of poly(ethylene glycol) in plasma protein fractions. *Anal Biochem* **107**: 60–63

Sung C, Van Osdol WW, Saga T, Neumann RD, Dedrick RL and Weinstein JN (1994) Streptavidin distribution in metastatic tumors pretargeted with a biotinylated monoclonal antibody: theoretical and experimental pharmacokinetics. *Cancer Res* **54**: 2166–2175

Veronese FM, Monfardini C, Caliceti P, Schiavon O, Scrawen MD and Beer D (1996) Improvement of pharmacokinetic, immunological and stability properties of asparaginase by conjugation to linear and branched monomethoxy poly(ethylene glycol). *J Control Release* **40**: 199–209

Wilchek M and Bayer EA (1988) The avidin–biotin complex in bioanalytical applications. *Anal Biochem* **171**: 1–32

Wilchek M and Bayer EA (1989) Avidin–biotin technology ten years on: has it lived up to its expectations? *Trends Biol Sci* **14**: 408–413

© Cancer Research Campaign 1998